# Towards an understanding of the information and support needs of surgical adolescent idiopathic scoliosis patients: a qualitative analysis

**DOI:** 10.1186/1748-7161-4-12

**Published:** 2009-05-08

**Authors:** Radha MacCulloch, Sandra Donaldson, David Nicholas, Joyce Nyhof-Young, Ross Hetherington, Doina Lupea, James G Wright

**Affiliations:** 1 The Hospital for Sick Children, Toronto, Ontario, Canada; 2 Toronto General Hospital, Toronto, Ontario, Canada; 3 Cancer Care Ontario, Ontario, Canada

## Abstract

**Background:**

Informed decision making for adolescents and families considering surgery for scoliosis requires essential information, including expected outcomes with or without treatment and the associated risks and benefits of treatment. Ideally families should also receive support in response to their individual concerns. The aim of this study was to identify health-specific needs for online information and support for patients with adolescent idiopathic scoliosis who have had or anticipate having spinal surgery.

**Methods:**

Focus group methodology was chosen as the primary method of data collection to encourage shared understandings, as well as permit expression of specific, individual views. Participants were considered eligible to participate if they had either experienced or were anticipating surgery for adolescent idiopathic scoliosis within 12 months, were between the ages of 10 and 18 years of age, and were English-speaking.

**Results:**

Two focus groups consisting of 8 adolescents (1 male, 7 female) and subsequent individual interviews with 3 adolescents (1 male, 2 female) yielded a range of participant concerns, in order of prominence: (1) recovery at home; (2) recovery in hospital; (3) post-surgical appearance; (4) emotional impact of surgery and coping; (5) intrusion of surgery and recovery of daily activities; (6) impact of surgery on school, peer relationships and other social interactions; (7) decision-making about surgery; (8) being in the operating room and; (9) future worries.

**Conclusion:**

In conclusion, adolescents welcomed the possibility of an accessible, youth-focused website with comprehensive and accurate information that would include the opportunity for health professional-moderated, online peer support.

## Background

Scoliosis surgery is the most common elective paediatric orthopaedic procedure. Patient decision making about surgery involves weighing the risks of surgery relative to families' perception of the current deformity and the possibility for future progression. Conflicting research regarding surgical outcomes and the risk-benefit ratio of spinal surgery for adolescent idiopathic scoliosis invites further consideration for patients and families [1,2]. Accordingly, the decision to undergo surgery is an important and difficult one for adolescent patients and their families. For those who elect to proceed, significant support is required throughout the decision-making process and surgical experience [3]. Thus, two essential and related requirements for patients considering spinal surgery are accessible and relevant information and ongoing support [3].

Failure to fully address patients' and families' health information needs is frequently noted in the literature across health conditions [4-11]. Given resource restrictions in the usual outpatient setting, clinicians are typically unable to completely satisfy these informational needs. As a result, many families turn to the internet to increase their knowledge and seek support. However, current online resources provide variable, conflicting and often untrustworthy information, so even if families can find relevant information; they are unsure which information to trust [12].

E-health, defined as the application of the internet and related technologies in health care to improve access, efficiency, effectiveness, and quality of care, has the potential to partially address the unmet needs of patients and their families and augment clinical relationships [13-20]. A key issue for the development of appropriate and useful web content is to determine the needs of the user. The purpose of this study is to determine: (1) the perceived information and support needs of adolescents with scoliosis; and (2) how these reported needs for information and support can be addressed using internet technology for the development of a web-based interactive intervention.

## Methods

Adolescent patients, selected from the orthopaedic clinic of The Hospital for Sick Children (SickKids), Toronto, were considered eligible to participate if they had either experienced or were anticipating surgery for adolescent idiopathic scoliosis (AIS) within 12 months, were between 10 and 18 years of age, and were English-speaking. Of the 20 potential participants identified as meeting eligibility criteria, 11 consented to participation, 6 refused to participate due to lack of interest and 3 could not be reached. The refusals were comparable to participants in regards to age (mean: 15 years of age: range 12–17 years of age), cultural background (22% African American, 78% Caucasian), and distance from hospital (mean: 50 km; range: 30–126 km).

A diverse sample of 11 participants who ranged in age (mean: 16 years of age; range: 10–18 years of age), sex, cultural background (9% Asian; 27% African American; 64% Caucasian) and distance from hospital (mean: 38 km; range: 5 km–113 km) participated in the study. Ethics approval from the Research Ethics Board at the Hospital for Sick Children (Toronto, Ontario) was obtained prior to study initiation. Informed consent was obtained from all participants prior to study participation and all participants were informed that focus group transcripts would be cleaned of identifying information to maintain anonymity and necessary steps would be taken to ensure participant privacy and confidentiality. All participants identified themselves to be regular computer and internet users. They identified keeping in touch with friends, playing online games, and researching school projects as the primary reasons for use of the internet.

The first focus group consisted of 5 participants (1 male and 4 female) and the second of 3 participants (all female). Three participants (1 male, 2 female) took part in one-on-one telephone interviews. Although the sample was predominately female, this number is reflective of the distribution of sex relative to AIS. Table 1 provides participant demographics and clinical data.

**Table 1:** Patient demographic & clinical data

**Patient**	**Sex**	**Age**	**Follow Up**
1	M	15	6 mo post-op

2	F	17	3 mo post-op

3	F	17	15 mo post-op

4	F	18	9 mo post-op

5	F	15	4 mo post-op

6	F	16	Pre-op

7	F	16	Pre-op

8	F	10	Pre-op

9	M	17	12 mo post-op

10	F	16	Pre-op

11	F	16	Pre-op

Focus groups were used as the primary method of data collection, as this forum encouraged both individual perspectives and the development of a shared understanding of the perceptions of adolescent patients [21]. One-on-one telephone interviews were held with those participants who were unable to attend focus groups due to scheduling conflicts or distance from hospital. Focus group size was chosen in accordance with the guidelines outlined by Merton et al. [22]. Focus groups were 85 to 110 minutes in length (mean = 97.5 minutes) and were facilitated by a research coordinator (MSW) with extensive focus group facilitation experience. A note-taker and observer were present to capture group dynamics and ensure that comments were not overlooked or misinterpreted. A semi-structured interview guide (see Appendix 1) was used to focus the discussion, while eliciting individual views and description of experiences [23]. An emergent website table of contents (see Appendix 2) and preliminary structural mock-up, based in part on the research team's prior experience and a thorough critical analysis of other websites and the literature, were also presented during focus groups and phone interviews to elicit feedback regarding content preferences and the flow and desired presentation of information and support.

Focus groups and telephone interviews were audio-recorded, transcribed verbatim and analyzed using NVivo  qualitative research software. Transcripts were subjected to content analysis, concept saturation and theme generation, using 'long interview' qualitative research techniques [24]. Key concepts, categories and emerging themes were inferred from participants' comments. Based on established techniques for demonstrating rigor in qualitative research, trustworthiness of findings was verified through: (i) team coding clarification and consensus; (ii) referential adequacy through sufficient inclusion of text quotes as interpretation verification; (iii) member checking, and (iv) peer debriefing among team members and appropriate clinical and web technology consultants [25].

## Results

Participants were actively engaged in focus group discussion and shared a range of perspectives. As adolescent patients, participants identified many unanswered questions unique to both their age and health condition and strongly endorsed the need for a customized, web-based informational and support resource. Based on the volume and poignancy of comments made, participants were concerned with: (1) recovery at home; (2) recovery in hospital; (3) post-surgical appearance; (4) emotional impact of surgery and coping; (5) intrusion of surgery and recovery of daily activities; (6) impact of surgery on school, peer relationships and other social interactions; (7) decision-making about surgery; (8) being in the operating room, and (9) future worries. See Table 2 for a summary of theme counts.

**Table 2:** Theme counts

**Theme**	# of Passages*	# of Documents**
- Recovery at home	48	5
- Pain control***	10	3

- Recovery in hospital	21	5

- Post-surgical appearance	20	5

- Emotional impact of surgery	18	5
- Coping***	12	4

- Impact on activities	14	4
- Instrumentation***	11	4

- School	13	4
- Social interactions***	13	4
- Friends***	11	2

- Making the decision to have surgery	11	4

- Being in the operating room	8	2

- Future worries	7	2

* # of Passages is the number of times that each theme was coded across all documents.

** # of Documents defined as the number of focus groups and interviews (max of 5) in which the theme emerged.

*** Some themes were grouped together when discussed here as they are inter-related; however each theme was coded (and counted) independently.

### Recovery at Home

Participants wanted to know about expected after-effects of scoliosis surgery including numbness, stiffness, sensitivity and restricted mobility, and also were interested in tips for easing recovery discomfort. Teens wanted information about how to protect the scar against infection, how to shower, especially in the first few weeks, and sun exposure. One adolescent who had had surgery explained, *"For the first month I had to keep a towel over it. Just because I wanted to – I don't know why, I just really wanted to – you were afraid it was going to sting and get infected" (Female participant)*.

Teens were concerned about insufficient pain control or conversely, dependence on painkillers. One adolescent had felt unprepared for dealing with the pain and discomfort: "*I don't think there was anything I could have done to make me more comfortable. But in terms of being prepared, I wasn't really prepared for the pain – even when you get through the pain, you're constantly uncomfortable" (Male participant)*. Increased tangible support from others, specifically family, was noted as a necessity in recovery; however this sudden dependence was also found to be a challenge for teens. One patient explained, *"...getting out of bed...just standing up; my Mom would basically have to pick me up and put me on my feet. You can't do anything by yourself, and even if it's the middle of the night and you want to get up and go to the bathroom, you need to call somebody, and they have to come and get you. It's not like the nurses who [were] always there, checking up on you every hour. You have to call your parents or something, and they're asleep. And yeah, it's harder to be at home, but at the same time it's nicer, because it feels better to get out of the hospital environment" (Female participant)*.

### Recovery in Hospital

Adolescents wanted to know the length of hospital stay, length of time they would be confined to the hospital bed, the layout and size of the hospital room and whether it would be shared or private, and the kinds of activities that would be available to pass the time while in-hospital. A post-operative patient recalled the importance of *"knowing when you can go home. They [the health care team] were like, 'Okay, you have to eat and then you can go home'. And I didn't want to eat. ... As soon as I ate one little piece of toast, they're like 'Okay, you can go home'. And I was like, 'That's all? Shouldn't you still be worried?' But it was perfectly fine. Like doctors do know what's best" (Female participant)*. Furthermore, for those participants who had undergone surgery, many explained that they would have liked to have known of the physical after-effects of being anaesthetized and medicated for pain control. For example, several experienced swelling, fever, and strange dreams.

### Post-Surgical Appearance

Post-surgery information needs concerning appearance revolved around changes to expect such as increased height, decreased rib prominence, and shoulder and hip balance. One teen wondered how a possible change in her appearance would affect the type of clothing she could wear: *"I just worry about if you can wear certain things. I know now I have problems dressing sometimes because of my hips. When I wear tighter, fitted shirts, it shows more obvious. But some people might be worried if their scar will stick out" (Female participant)*.

### The Emotional Impact of Surgery and Coping Strategies

The emotional impact of surgery in pre- and post-operative periods and how patients coped with their surgery-related thoughts, feelings, and concerns emerged as a significant topic of discussion in all focus groups and telephone interviews. Prior to surgery, adolescents described experiencing a range of emotions, including being "*nervous*", "*excited*", "*anxious*" and described "*just wanting to get it done*". To cope with their feelings and thoughts pre-surgery, adolescents described talking and spending time with family members or friends, making jokes about surgery, reading, listening to music, and educating themselves about the surgery itself. Post-surgery, participants described feelings of irritability, depression, frustration, and anxiety related to being left alone. One teen described transient feelings of regret post-surgery: *"I think it was the fact that I was just sitting in that hospital bed and was thinking 'Why did I do this?' From that perspective [of being temporarily immobile] you really don't see the reason why you did it" (Male participant)*. Patients mentioned the importance of getting up and walking, having visitors in hospital and at home, and going out of the house to spend time with peers as methods of coping during the post-surgical recovery period.

### Intrusion of Surgery on Daily Activities

Participants were eager to know how surgery and recovery would affect their daily activities such as sports including yoga, basketball, swimming, and ballet, and travel. The issue of whether the implanted hardware would cause difficulty with metal detection devices at airports and other secure locations was raised.

### Impact of Surgery on School, Peer Relationships and Other Social Interactions

Patients also wanted to know how surgery would influence school attendance, social interactions, friendships, and dating relationships. Adolescents were concerned about responding to possible overreactions of others, and were worried that others might stare at their scars or treat them differently. In addition, they worried about being able to maintain friendships and relationships. One participant explained that while in hospital, *"You don't know how people are going to react to the fact that you're there with no makeup, with your hair all greasy. And you feel all gross, and you feel bad about yourself. And everyone else is going to think that I am ugly. And they're not going to want to stay there with me, because I'm boring" (Female participant)*. Patients described relationships with peers as being potentially both positive and negative. Adolescents described support from peers as helping them cope both pre- and post-surgery. In fact, one teen felt, "*If I didn't have the friends who came to visit me, I would have been screwed. I wouldn't have recovered as fast as I did. I know it sounds cheesy, but support helps you recover faster" (Female participant)*. Conversely, adolescents were distressed when friends were not supportive of their decision to have surgery or did not visit them during recovery. "*I remember like my friends never showed up, and I was so cheesed; like they wouldn't even call" (Male participant)*.

### Decision-making Related to Surgery

In general, the possibility of future progression should they not proceed with surgery ultimately influenced participants' decision to undergo surgery. Further, teens identified a desired change in appearance and a wish to "*look all the more normal*" as influencing their decision. These adolescents valued being able to make the decision independently, but with the support of others, and they wanted to know how others were diagnosed and came to their decisions to have surgery.

### In the Operating Room

The adolescents were curious about instrumentation. They grappled with questions of whether they would feel the internal implants. Pre-operative adolescent participants also wanted to know what to expect in the operating room on the day of surgery. Specifically, patients wanted information about undergoing anaesthesia, including the time required to induce anaesthetization and if they would feel anything while unconscious. They were interested in a description of the operating room and whether they could bring a personal item into the operating room.

### Future Concerns

Adolescents were worried about long-term surgical outcomes including effects on employment, pregnancy, and the possibility of future back discomfort or problems. Although these long-term concerns were identified, they were the least prominent of the themes, emerging the fewest times in the analysis.

### Suggestions for Web-based Interaction

Adolescents identified changes or areas for improvement in the content, format and organization of the website mock-up. Adolescents overwhelmingly wanted the website to include an interactive or personalized component. For example, they felt that many of their informational needs could be met through online or virtual "social networking". Specifically, posted biographies or online discussion forums were selected as their preferred methods of online interaction. Participants wanted to share their own stories. This demonstrates the potential importance of social comparison as a coping method and the therapeutic effects of sharing one's personal story with others. Teens suggested including photos of real people who had undergone surgery for scoliosis and photos demonstrating outcome variation of curvature and scarring. One teen, who had been worried about how the surgery would impact his mobility and extracurricular activities, suggested adding "*pictures of people actually doing things after surgery*", as this would have helped him stay positive and hopeful.

Participants identified that just as the information on the website must be reflective of the unique needs of adolescent surgical patients, the website's design and organization should also be tailored to this particular group. The information should be clear and concise, and presented in a colorful interface with design elements reflecting teenage tastes. It was recommended that information be organized according to differing needs potentially dictated by gender, age, and type of user – patient or parent. As one participant explained, "*I think it's [the site] more for the kids, because the parents obviously want to know stuff, but the kids are the ones actually going through it. So they have more questions than parents, I guess*".

## Discussion

Information about scoliosis surgery is required by patients and families for shared decision-making. Adolescents and families considering scoliosis surgery clearly seek specific information, including the expected outcomes with or without surgery and the potential risks and benefits of treatment. A minority of families seek either complete control over treatment decisions or abdicate decision-making control to their physicians [26]. The majority of patients want to share decision-making with their physicians [27]. Clearly, any information provided to patients and families as a prerequisite to effective decision making must be complete, accurate, and individualized.

Although the themes emerging from the focus groups were for the most part expected, the intensity of concern underlying certain themes was unexpected. Post-operative patients expressed serious concern about addiction to analgesics and worried about their self-weaning process during their recovery at home. Patients experienced fear and anxiety the first four to six weeks at home including fear of being alone and fear of waking in the night when family members were asleep. Patients also expressed disappointment in friends and dating partners who were unable to support them during their recovery. These teens indicated an increased emotional dependence on parents during this time and stressed the need for social support during the first month or two following hospital discharge. Based on these findings, an interactive, peer support component should be an integral part of a website designed for adolescent surgical candidates. Although these adolescents would like access to comprehensive information about surgery, they want to hear about it from the perspective of other adolescents who have undergone surgery themselves. An online discussion forum or posted biographies would give teens the opportunity to learn about surgery through the experiences of others, while giving those who have had surgery the opportunity to share their stories with others.

E-health has the potential to partially address the unmet needs of the family. Web-based information can be streamlined according to particular case profiles and population needs [28]. Individuals can access this information whenever and however they wish [13]. Research suggests that the impact of illness-related websites is enhanced by providing full and in-depth information [12] combined with the opportunity for interaction [13,29]. This may be particularly true of adolescents who tend to be relatively active computer users [30] and seldom participate extensively during health-care interactions. To enhance the effectiveness of e-health applications, web-based information and support should be reflective of patients and families information needs and their preferences for content and presentation style.

This study has several potential limitations. First, focus group and interview participants were pre- and post-operative patients from one hospital; and issues in other centres or regions may vary. However, the findings were reasonably consistent across focus groups and interviews, suggesting that the major issues relevant to designing an effective website were identified. Second, the sample size was relatively small. It was, however, representatively diverse. A larger and more diverse sample could uncover a greater range of information needs. However, the participants in this study provided what appeared to be a comprehensive description of needs for information, support features such as a discussion forum, individual peer perspectives and stories, and user-focused design elements. These data are guiding the design of a website for adolescents undergoing surgery and their families. The website will continue to evolve based on feedback received from its users.

## Conclusion

In conclusion, adolescents welcomed the possibility of an accessible, youth-focused website with comprehensive and accurate information that would include the opportunity for health professional-moderated, online peer support.

## Competing interests

The authors declare that they have no competing interests.

## Authors' contributions

RM and SD completed data collection, data analysis and drafted the manuscript. DN participated in the development of study design and reviewed and edited drafts of the manuscript. RH developed the emergent website table of contents and preliminary structural mock-up of the website and reviewed and edited drafts of the manuscript. JNY and DL reviewed and edited drafts of the manuscript. JGW conceived of the study, participated in its design and coordination and reviewed and edited drafts of the manuscript. All authors reviewed and approved the final manuscript.

## Appendix 1 – interview guide

What are your needs?

• What do children with scoliosis need to help them with this condition?

• What do parents of children with scoliosis need to help them with this condition?

• What do others in the family need to help them with this condition and its impact on the family?

Have you used the Internet to find out about scoliosis?

• Have you used a website before? If so, what did you like about it, what did you not like?

• How could online or web-based sources be helpful to families?

• What would need to be in a website for it to be most helpful to you?

• What would prompt you to use the computer for information or support?

• What would prompt your family to use the computer for information or support?

• What are the limitations to accessing the computer?

What would be an ideal website?

• If you were to design a table of contents for a website, what would you include?

• We've designed possible areas that could be addressed on a website? Could we review these? [Note: Present potential table of contents]

• For each identified area, could you indicate if you believe this idea is relevant, important, and clear

• Can you comment on the ordering of areas in the table of contents? What changes should be made. Explain.

## Appendix 2 – information architecture of the scoliosis website

### Scoliosis information architecture: Teen site

Is Scoliosis Surgery for You?

• Ottawa Personal Decision Guide

• How did other Teens Decide?

• Teens, Parents, Surgeons: Who's Thinking What?

Your Surgery

• Preparing for Surgery

◦ What to Avoid Before Surgery

◦ What to do Before Surgery

◦ Arriving at the Hospital

• During Surgery

• After Surgery

◦ Monitoring

◦ Hospital Equipment

◦ Personal Care

◦ Taming Your Hospital Stay

◦ Leaving the Hospital

◦ Needing a Brace after Surgery

• FAQs for the Surgeons

The First Few Months after Surgery

• Your Incision Scar, Numbness, and Pain

• Changes in Body Shape and Back Movement

• Coping with the Changes

Coping with Your Emotions

• Your Emotions: Before Surgery

• Your Emotions: In the Hospital

• Your Emotions: At Home

Coping with Your Family and Others

• Your Boyfriend or Girlfriend

• Your Friends

• School

• Work

• Who Else Needs to Know?

Personal Stories

• M's Story

• K's Story

• K's Story

• A's Story

### Scoliosis information architecture: Parent site

About Scoliosis

• What Causes Scoliosis?

• Who Gets Scoliosis?

• What Does Scoliosis Look Like?

• Scoliosis and Emotional Issues

• Does Scoliosis Cause Pain?

• Does Scoliosis Affect the Heart and Lungs?

• References

Understanding Diagnosis

• The Physical Exam

• X-ray

• CT Scan

• MRI

• The Health Care Team

• Informed Consent

• References

Treatment

• What Happens if Scoliosis is not Treated?

• Immediate Risks of Surgery

◦ Pros of Having or not Having Surgery

◦ Cons of Having or not Having Surgery

• Surgery in Boys

• Ottawa Personal Decision Guide

• Teens, Parents, Surgeons: Who's Thinking What

• The Health Care Team

• Preparing for Surgery

◦ What to Avoid Before Surgery

◦ What to do Before Surgery

◦ Arriving at the Hospital

• During Surgery

• After Surgery

◦ Monitoring

◦ Hospital Equipment

◦ Personal Care

◦ Leaving the Hospital

◦ Needing a Brace after Surgery

• FAQs for Surgeons

• Helping your Teen Cope

• Informed Consent

• References

At Home

• Recovering from Surgery

◦ Pain Management

◦ Returning to Regular Activities

◦ Clinic Visits

• Complications to Watch for

• References

Looking Ahead

• What to Expect in Adulthood

• The Future of Scoliosis

• Resources

• References
